# PFClust: an optimised implementation of a parameter-free clustering algorithm

**DOI:** 10.1186/1751-0473-9-5

**Published:** 2014-02-04

**Authors:** Khadija Musayeva, Tristan Henderson, John BO Mitchell, Lazaros Mavridis

**Affiliations:** 1 School of Computer Science, University of St Andrews, North Haugh, St Andrews, Scotland KY16 9SX, UK; 2 EaStCHEM School of Chemistry and Biomedical Sciences Research Complex, University of St Andrews, North Haugh, St Andrews, Scotland KY16 9ST, UK

**Keywords:** Clustering, Cluster analysis, Number of clusters

## Abstract

**Background:**

A well-known problem in cluster analysis is finding an optimal number of clusters reflecting the inherent structure of the data. PFClust is a partitioning-based clustering algorithm capable, unlike many widely-used clustering algorithms, of automatically proposing an optimal number of clusters for the data.

**Results:**

The results of tests on various types of data showed that PFClust can discover clusters of arbitrary shapes, sizes and densities. The previous implementation of the algorithm had already been successfully used to cluster large macromolecular structures and small druglike compounds. We have greatly improved the algorithm by a more efficient implementation, which enables PFClust to process large data sets acceptably fast.

**Conclusions:**

In this paper we present a new optimized implementation of the PFClust algorithm that runs considerably faster than the original.

## Introduction

Cluster analysis [1] comprises methods designed to find structure in a dataset. Data can be divided into clusters that help us understand the problem domain, inform ongoing investigation, or form input for other data analysis techniques. Clustering methods [2-7] attempt to find such clusters based only on the known relationships between the data objects. This distinguishes them from supervised data analysis approaches, such as classification methods [8] that are provided with right and wrong answers to guide their data analysis. One of the main challenges introduced by the lack of class labels is determining an optimal number of clusters that reflect the inherent structure present in the data. Exhaustive cluster enumeration becomes impractical as the size and dimensionality of the data grow. We have developed a novel clustering technique called PFClust [9] that automatically discovers an optimum partitioning of the data without requiring prior knowledge of the number of clusters. PFClust is also immune to the enumeration problem introduced by high-dimensional data, since it relies on a similarity matrix. Here we give a brief overview of the algorithm and its applications, and present a new efficient implementation.

## PFClust

PFClust is based on the idea that each cluster can be represented as a non-predetermined distribution of the intra-cluster similarities of its members. The algorithm partitions a dataset into clusters that share some common attributes, such as their minimum expectation value and variance of intra-cluster similarity. It is an agglomerative algorithm, starting with separated objects and progressively joining them together to form clusters. The algorithm attempts clustering using 20 threshold values, chosen using a random sampling technique, and then uses the Silhouette width to select which of the clusterings best describes the input dataset.

## Method

PFClust consists of two steps: threshold estimation and clustering. The threshold estimation procedure randomly splits the given dataset into clusters 1000 times and records the expectation value of the intra-cluster similarities between members of the same cluster. Twenty threshold values from the top 5% of the distribution of mean intra-cluster similarities are selected as a representative range of possible thresholds, and are fed into the subsequent clustering procedure. The clustering step of the algorithm is computationally more intensive than the threshold estimation step, and its complexity is *O(kn*^*2*^*)*, where *k* is the number of clusters and *n* is the number of elements in the dataset. This is also the overall complexity of the PFClust algorithm.

Without changing the computational complexity of the algorithm we have re-implemented it in the same programming language as the original (Java) with a careful selection of data types and appropriate bookkeeping, as the majority of operations are performed inside loops and involve intensive data structure manipulation. We have also taken advantage of the independence of the 20 iterations of the clustering procedure and executed them in parallel.

## Performance evaluation

We compare the performance of the original [9] and new PFClust implementations by measuring execution time on the following configuration:

•Hardware: 2.2 GHz Intel(R) Core(TM) i5-3470S CPU @ 2.90 GHz, 8.00 GB RAM

•Operating system: Scientific Linux release 6.3 (Carbon)

•JVM: 1.6.0_45-b06

The running times of each step of the PFClust algorithm (threshold estimation, clustering, and the main iteration that combines these steps) have been evaluated separately. Each step and the main iteration were executed 10 times, and average run times were obtained. The first step of the algorithm, involving random number generation, was initialized with the same seed in both implementations to keep the number of calculations approximately constant. The clustering was executed with the same set of threshold values for each dataset in both programs. The main iteration carried out the randomization step with the same seed and the clustering procedure with the same thresholds.

The performance improvement of the new implementation is primarily due to the representation of the similarity matrix and cluster objects. The old implementation used string objects as row and column names and looked up values in the similarity matrix based on these names. The names were stored in a vector, and searching for an element in a vector data type is *O(n)* where *n* is the number of elements. Many operations involved two nested loops to search for the corresponding row and column names, which resulted in *O(n*^*2*^*)* behaviour. The cluster objects in the old implementation were also backed by vectors of strings and involved intensive computations. There was an additional performance overhead related to synchronization of vectors, producing an overall performance bottleneck. The new implementation utilizes a two dimensional array of primitives to represent the similarity matrix and an ArrayList data type to represent cluster objects. The values are retrieved from the array or ArrayList based on the index, a constant time operation. Unlike the old implementation, the new code utilizes bookkeeping with HashSet and ArrayList data types, where applicable, to decrease the number of operations inside the loops. In the threshold estimation step, the data are now sorted before retrieving the required values from the array, whereas the values were selected in a brute-force fashion in the old implementation.

**Figure 1:**
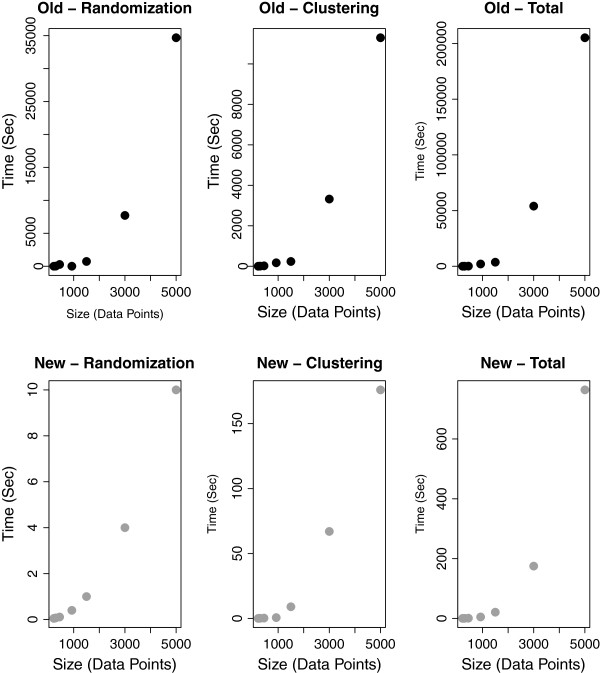
**Execution times.** Comparison of the execution times between the original (black, top row) and new (grey, bottom row) implementations, averaged over the seven datasets from [9]. The different steps of the algorithm (Randomization, Clustering and Total Execution time) are shown from left to right. The combined process of randomization and clustering has to be run four times (or occasionally more [9]), the totals given here include these repetitions.

The evaluation results (Figure 1) show that the execution times are greatly improved. The clusterings resulting from the two implementations agree closely, with a very high average Rand Index [10] of 0.985 over the seven datasets from [9].

## Applications

The previous implementation has already been successfully used for biologically related problems with very promising results [9]. A set of protein domains taken from CATH [11] were clustered using a spherical polar Fourier shape-based representation [12,13]. PFClust proposed 11 protein families and one singleton domain, whereas CATH clusters them into 11 families. While CATH superfamilies are based on protein structures that share a common fold, structures in the same superfamily might differ considerably [13]. Hence, approaches like PFClust could be used to refine the current families and to identify interesting outliers or problematic cases.

PFClust has also been successfully used to cluster a large number of small molecular structures [14]. ChEMBL [15] holds information on over 1,000,000 compounds and groups them into families according to their experimental bioactivities. These families were individually clustered using PFClust to create “refined” families which significantly improved the precision of our protein target predictions.

## Conclusion

An efficient implementation of PFClust enabled us to run the program on all our synthetic datasets [9] acceptably fast. It processes the largest data set (5000 2D Vectors) in minutes, while the original implementation took several days. This new implementation can be now used effectively, not only for small datasets (≤ 1500) as previously shown, but also for larger ones (≥ 5000).

## Competing interests

The authors have received funding from WADA. Other than this sponsorship, the authors declare no conflict of interest.

## Authors’ contributions

KM developed the software. KM and LM designed the software. LM conceived the original idea. TH, JBOM and LM supervised the project. All authors participated in the drafting of the manuscript. All authors have read and approved the final manuscript.

## References

[B1] Jain AK Murty MN Flynn PJ Data clustering: a review ACM Comput Surv 1999 31 264 323 10.1145/331499.331504

[B2] Lance BGN Williams WT A general theory of classificatory sorting strategies 1: hierarchical systems Comput J 1967 9 373 380 10.1093/comjnl/9.4.373

[B3] Jain AK Data clustering: 50 years beyond K-means Pattern Recogn Lett 2010 31 651 666 10.1016/j.patrec.2009.09.011

[B4] Ester M Kriegel HP Sander J Xu X A density-based algorithm for discovering clusters in large spatial databases with noise Proc 2nd Int Conf Knowl Discov Data Min 1996 KDD-96 226 231

[B5] Wei C Empirical comparison of fast clustering algorithms for large data sets Expert Syst Appl 2003 24 351 363 10.1016/S0957-4174(02)00185-9

[B6] Fraley C Raftery AE Model-based clustering, discriminant analysis, and density estimation J Am Stat Assoc 2002 97 611 631 10.1198/016214502760047131

[B7] Kaufman L Rousseeuw PJ Finding Groups in Data: An Introduction to Cluster Analysis 1990 New York: Wiley

[B8] Finley T Joachims T Supervised clustering with support vector machines ICML '05 Proceedings of the 22nd International Conference on Machine Learning 2005 217 224

[B9] Mavridis L Nath N Mitchell JBO PFClust: a novel parameter free clustering algorithm BMC Bioinformatics 2013 14 213 10.1186/1471-2105-14-213 23819480 PMC3747858

[B10] Rand WM Objective criteria for the evaluation of clustering methods J Am Stat Assoc 1971 66 846 850 10.1080/01621459.1971.10482356

[B11] Cuff AL Sillitoe I Lewis T Redfern OC Garratt R Thornton J Orengo CA The CATH classification revisited-architectures reviewed and new ways to characterize structural divergence in superfamilies Nucleic Acids Res 2009 37 D310 D314 10.1093/nar/gkn877 18996897 PMC2686597

[B12] Mavridis L Ritchie DW 3D-Blast: 3D protein structure alignment, comparison, and classification using spherical polar Fourier correlations Pac Symp Biocomput 2010 2010 281 292 19908380

[B13] Mavridis L Ghoorah AW Venkatraman V Ritchie DW Representing and comparing protein folds and fold families using three-dimensional shape-density representations Proteins: Struct, Funct Bioinform 2011 80 530 545 10.1002/prot.23218 22081520

[B14] Mavridis L Mitchell JBO Predicting the protein targets for athletic performance-enhancing substances J Cheminform 2013 5 31 10.1186/1758-2946-5-31 23800040 PMC3701582

[B15] Gaulton A Bellis LJ Bento PA Chambers J Davies M Hersey A Light Y McGlinchey S Michalovich D Al-Lazikani B Overington JP ChEMBL: a large-scale bioactivity database for drug discovery Nucleic Acids Res 2012 40 D1100 D1107 10.1093/nar/gkr777 21948594 PMC3245175

